# Identification and characterization of SSEA-3⁺/CD105⁺ cells in adipose tissue of rabbits, dogs, and cats: insights into a Muse-like cell population

**DOI:** 10.1016/j.vas.2026.100917

**Published:** 2026-10-25

**Authors:** María Gemma Velasco-Martínez, Belén Cuervo, Emma Martins, Rocío Colomer-Selva, Maria Descalzo-Navarro, Beatriz Mena-Moros, Deborah Chicharro, José María Carrillo, Joaquín Jesús Sopena, Mónica Rubio, Katiuska Satué

**Affiliations:** Bioregenerative Medicine and Applied Surgery Research Group, Department of Animal Medicine and Surgery, CEU Cardenal Herrera University, CEU Universities, Alfara del Patriarca, 46115, Valencia, Spain

**Keywords:** Muse-like cells, Characterization, Adipose tissue, Stem cells, Rabbit, Dog, Cat

## Abstract

Muse cells have gained interest for their pluripotency, stress tolerance, targeted migration to injury sites, and non-tumorigenic nature. Although they can be isolated as a SSEA-3⁺/CD105⁺ population, their characterization across animal species remains limited. This study aimed to characterize adipose-derived SSEA-3^+^/CD105^+^ cells from rabbits, dogs, and cats, and to assess the expression of Sox2, Nanog, and Oct3/4 exclusively in rabbit SSEA-3+/CD105+ cells. Samples were collected from inguinal fat in rabbits, falciform ligament fat in dogs, and mesenteric fat in cats. Adipose-derived stem cells (ADSCs) were cultured prior to quantification of SSEA-3 and CD105 by immunofluorescence. Subsequently, rabbit ADSCs underwent immunomagnetic purification to obtain SSEA-3^+^/CD105^+^ cells, which were then assessed for Sox2, Nanog, and Oct3/4 expression by immunofluorescence. SSEA-3 and CD105 expressions were detected at levels of 54.84±7.34%, 39.93±9.58% and 50.61±16.67% in rabbits, dogs and cats, respectively. Most purified rabbit SSEA-3^+^/CD105^+^ cells were Nanog+ (87.65±5.16%), Oct3/4+ (88.96±1.55%) and Sox2+ (89.15±2.84%). This study identifies an SSEA-3⁺/CD105⁺ cell population in adipose tissue from rabbits, dogs, and cats that exhibits immunophenotypic characteristics consistent with a Muse-like population and reports the expression of the pluripotency-associated markers Nanog, Oct3/4, and Sox2 in rabbit SSEA-3⁺/CD105⁺ cells. Adipose tissue emerges as a practical and accessible source of SSEA-3⁺/CD105⁺ cells in veterinary species. These findings suggest that species-, tissue-, and methodology-related factors may contribute to the variability observed in the proportion of SSEA-3⁺/CD105⁺ cells and provide a basis for future studies aimed at the functional characterization of this population and its potential relevance in regenerative medicine.

## Highlights

• Muse-like cells were identified as SSEA-3^+^/CD105^+^ cells in rabbits, dogs, and cats.
• SSEA-3^+^/CD105^+^ cells in rabbits, dogs and cats: 54.84±7.34, 39.93±9.58, 50.61±16.67% of ADSCs.
• Sox2, Nanog, and Oct3/4 expression was assessed in purified rabbit SSEA-3^+^/CD105^+^ cells.
• Purified rabbit SSEA-3^+^/CD105^+^ cells showed high expression of Nanog, Oct3/4, and Sox2.
• Adipose tissue represents an accessible source of SSEA-3^+^/CD105^+^ cells in veterinary medicine.

## 1 Background

Regenerative medicine has become increasingly relevant in veterinary practice, providing new therapeutic options for osteoarthritis, organ fibrosis, and neurological disorders in companion animals(Aeri et al., 2025). Among the most widely studied approaches are mesenchymal stem cells (MSCs) and platelet-rich plasma (PRP)(Chang et al., 2025). MSCs are multipotent cells capable of self-renewal and differentiation into several specialized cell types, including osteoblasts, chondrocytes, and adipocytes(Pittenger et al., 1999).

In 2010, Dezawa’s group identified within MSC populations a small subset of endogenous pluripotent stem cells termed Muse (multilineage-differentiating stress-enduring) cells(Kuroda et al., 2010). Muse cells can spontaneously differentiate into derivatives of the three germ layers(Kuroda et al., 2010) migrate to damaged tissues via the sphingosine-1-phosphate (S1P)–S1PR2 axis(Alanazi et al., 2023; Yamada et al., 2018), and exhibit high stress tolerance(Alessio et al., 2016; Alessio et al., 2018; Kuroda et al., 2010) as well as anti-apoptotic, anti-fibrotic and anti-inflammatory properties(Alanazi et al., 2023; Shono et al., 2021).

Unlike embryonic stem cells (ESCs) or induced pluripotent stem cells (iPSCs), Muse cells can be isolated from adult tissues—including bone marrow, adipose tissue, and skin—without gene induction(Wakao et al., 2011). They express pluripotency factors (Oct3/4, Sox2, Nanog) at lower levels than ESCs, and are non-tumorigenic and immune-tolerant, supporting their potential for allogeneic applications. Muse cells have shown therapeutic benefits in models of myocardial infarction(Castillo et al., 2023; Yamada et al., 2018; Yamada et al., 2022), stroke(Abe et al., 2020; Yamauchi et al., 2015), hepatic fibrosis(Iseki et al., 2017) and osteochondral defects(Mahmoud et al., 2017), among other diseases.

Although stage-specific embryonic antigen-3 (SSEA-3) has been widely used as a marker for Muse cell isolation, additional mesenchymal markers, such as CD105, have also been investigated to further characterize these cells in different tissue sources(Kuroda et al., 2010). In the present study, the combined expression of SSEA-3 and CD105 was evaluated to identify an SSEA-3^+^/CD105^+^ cell population exhibiting immunophenotypic characteristics consistent with a Muse-like population in adipose-derived cell cultures from different mammalian species.

Besides its use as a marker for Muse cell identification, SSEA-3 may also play a biological role in stem cell regulation. Previous studies have suggested that SSEA-3 is involved in FGF2-dependent self-renewal, clonogenicity, maintenance of stemness, and lineage commitment, indicating that this glycolipid may contribute to the biological properties of pluripotent-like stem cell populations rather than functioning solely as a passive surface marker (Aprile et al., 2023; Lee et al., 2025).

Muse cells have been described in several species—including mouse(Aprile et al., 2021; Nitobe et al., 2019), rat(Sun et al., 2020), rabbit(Yamada et al., 2018), swine(Iseki et al., 2021), goat(Yang et al., 2013), sheep(Castillo et al., 2023), horse(Lee, S. et al., 2025), dog and cat(Chen et al., 2026; Wakao et al., 2026), although the available evidence remains heterogeneous. Few studies have focused on adipose-derived Muse cells, often assessing only a limited set of markers. Moreover, most research has centered on bone marrow-derived Muse cells(Velasco et al., 2023), leaving important gaps regarding their abundance and characteristics in adipose tissue, as well as the influence of adipose tissue depot and isolation methodology on Muse cell yield.

Given the growing relevance of adipose-derived stem cells (ADSCs) in veterinary regenerative medicine, further studies are needed to characterize Muse cells across species and adipose depots and to assess their expression of pluripotency markers such as Nanog, Sox2, and Oct3/4.

The aim of this study was to identify an SSEA-3+/CD105+ cell population in rabbit, dog, and cat adipose tissue by immunofluorescence and to evaluate the expression of the pluripotency-associated markers Sox2, Nanog, and Oct3/4 in a purified rabbit SSEA-3+/CD105+ cell population.

## 2 Methods

### 2.1 Ethical approval

All procedures were approved by the Animal Ethics and Experimentation Committee of CEU Cardenal Herrera University and by the competent authority (authorization 2023-VSC-PEA-0242). Owners of dogs and cats provided written informed consent.

### 2.2 Rabbit adipose tissue samples collection

Samples were obtained from seven healthy female New Zealand rabbits, aged 7 months and weighing approximately 4 kg. A complete physical examination was performed before sedation. Sedation was induced with intramuscular administration of dexmedetomidine 0.01 mg/kg (Dexdomitor®, Orion Corporation, Finland), ketamine 20 mg/kg (Ketamidor®, VetViva Richter GmbH, Austria), and morphine 5 mg/kg (Morphine B. Braun, B. Braun Medical, Germany). After sedation, a 24G intravenous catheter (Vasofix® Certo, B. Braun Medical, Germany) was placed in the marginal ear vein, and the inguinal region was shaved and aseptically prepared.

Anesthesia was induced with intravenous propofol administered to effect (Propofol Lipuro®, B. Braun Medical, Germany), and maintained with inhaled sevoflurane (SevoFlo®, Zoetis, USA) vaporized in oxygen.

With the animal under anesthesia, adipose tissue samples were collected from inguinal fat under strict sterile conditions. Approximately 15 grams of sample were placed in 50 mL Falcon tubes containing 25 mL of high-glucose Dulbecco’s Modified Eagle Medium (DMEM) without L-glutamine, sodium pyruvate, nor 4-(2-hydroxyethyl)-1-piperazineethanesulfonic acid (HEPES) (DMEM 1X, Gibco™, Thermo Fisher Scientific, USA), supplemented with 1% penicillin/streptomycin/amphotericin B (Antibiotic-Antimycotic (100X), Gibco™, Thermo Fisher Scientific, USA). Following the laboratory’s instructions, samples were kept at room temperature until arrival at the laboratory.

After sample collection, euthanasia was performed intraoperatively by intravenous administration of sodium pentobarbital at a dose of 140 mg/kg (Dolethal, Vetoquinol, France).

### 2.3 Canine adipose tissue samples collection

Samples were obtained from three healthy, adult, female dogs that were brought to the Veterinary Hospital at CEU Cardenal Herrera University for elective ovariectomy. Only animals ASA I with normal physical examination, blood count, and biochemical analysis results were included. Sedation was performed by intramuscular administration of dexmedetomidine 5 μg/kg (Dexdomitor®, Orion Corporation, Finland), methadone 0.3 mg/kg (Semfortan®, Dechra, United Kingdom), and midazolam 0.1 mg/kg (Midazolam Normon, Normon, Spain). After sedation, a 20G intravenous catheter (Vasofix® Certo, B. Braun Medical, Germany) was placed, and the abdominal region was shaved and prepared aseptically.

Anesthesia was induced with intravenous propofol administered to effect (Propofol Lipuro®, B. Braun Medical, Germany), and maintained with inhaled sevoflurane (SevoFlo®, Zoetis, United States).

With the animal under anesthesia, adipose tissue samples were collected from the falciform ligament under strict sterile conditions using a laparotomic approach. Between 10 and 15 grams of sample (depending on the size and body condition of the animal) were placed in 50 mL Falcon tubes containing 25 mL of high-glucose DMEM without L-glutamine, sodium pyruvate, nor HEPES (DMEM 1X, Gibco™, Thermo Fisher Scientific, USA), supplemented with 1% penicillin/streptomycin/amphotericin B (Antibiotic-Antimycotic (100X), Gibco™, Thermo Fisher Scientific, USA). Samples were kept at room temperature until arrival at the laboratory.

As post-surgical treatment for ovariectomy, methadone was prescribed at 0.2 mg/kg IV (Semfortan®, Dechra, United Kingdom) every 4 hours with pain assessed using the Glasgow Composite Measure Pain Scale, and meloxicam at 0.2 mg/kg IV (Meloxidolor®, Dechra, United Kingdom) perioperative and 0.1 mg/kg PO SID (Metacam®, Boehringer Ingelheim Vetmedica GmbH, Germany) for the following 3 days.

### 2.4 Feline adipose tissue samples collection

Samples were obtained from three young, healthy adult cats that were brought to the Veterinary Hospital at CEU Cardenal Herrera University for elective ovariectomy. Only animals with normal physical examination, blood count, and biochemical analysis results were included. For sedation, intramuscular administration of dexmedetomidine 10 μg/kg (Dexdomitor®, Orion Corporation, Finland), ketamine 1 mg/kg (Ketamidor®, VetViva Ritcher GmbH, Austria), and methadone 0.3 mg/kg (Semfortan®, Dechra, United Kingdom) were administered. After sedation, a 22G intravenous catheter (Vasofix® Certo, B. Braun Medical, Germany) was placed, and the abdominal region was shaved and prepared aseptically.

Anesthesia was induced with alfaxalone 5 mg/kg administered intravenously (Alfaxan®, Zoetis, United States), and maintained with inhaled sevoflurane (SevoFlo®, Zoetis, United States).

With the animal under anesthesia, adipose tissue samples were collected from mesenteric fat under strict sterile conditions using a laparotomic approach. Between 10 and 15 grams of sample (depending on the body condition of the animal) were placed in 50 mL Falcon tubes containing 25 mL of high-glucose DMEM without L-glutamine, sodium pyruvate, nor HEPES (DMEM 1X, Gibco™, Thermo Fisher Scientific, USA), supplemented with 1% penicillin/streptomycin/amphotericin B (Antibiotic-Antimycotic (100X), Gibco™, Thermo Fisher Scientific, USA). Samples were kept at room temperature until arrival at the laboratory.

As post-surgical treatment for ovariectomy, methadone was prescribed at 0.2 mg/kg (Semfortan®, Dechra, United Kingdom) every 4 hours with pain assessed using the Glasgow Composite Measure Pain Scale, and meloxicam at 0.1 mg/kg SC (Meloxidolor®, Dechra, United Kingdom) perioperative and 0.05 mg/kg PO the following day (Metacam®, Boehringer Ingelheim Vetmedica GmbH, Germany).

### 2.5 Primary cell culture of adipose-derived stem cells (ADSCs) from adipose tissue samples

ADSCs were isolated and expanded at Leitat Managing Technologies (Barcelona, Spain). Briefly, 9–10 g of adipose tissue was washed repeatedly with PBS containing 5% penicillin/streptomycin/amphotericin B. Samples were minced using surgical scissors and digested with 0.2% collagenase type I (Sigma-Aldrich®, Germany), sterile-filtered through a 0.22 µm filter (Millipore®, Germany), at 37°C for 1 hour. After digestion, it was filtered with a 100µm filter (Millipore®, Germany) and mature adipocytes were eliminated from the stromal vascular fraction (SVF) by centrifugation at 600g for 10 minutes.

The resulting pellet was resuspended in high-glucose DMEM (HyClone™, Cytiva, USA) supplemented with 10% fetal bovine serum (FBS) (Gibco™, Thermo Fisher Scientific, USA) and 1% penicillin/streptomycin/amphotericin B (HyClone™, Cytiva, USA) and cultured at 37°C in a humidified atmosphere with 5% CO₂ at a density of 7.5 × 10³ cells/cm². Cells were expanded until reaching 80–90% confluence and subcultured up to passages 3–5.

### 2.6 Immunocytofluorescence analysis of CD105 and SSEA-3 in rabbit ADSCs

ADSCs were seeded onto coverslips (Mattek, USA) until reaching 50–60% confluence and then washed with PBS (HyClone™, Cytiva, USA) and fixed with 4% paraformaldehyde (Thermo Fisher Scientific, USA) for 10 minutes at room temperature. After three PBS washes, coverslips were blocked with PBS + 3% bovine serum albumin (BSA) (Sigma-Aldrich®, Germany) for 30 minutes.

Primary antibodies against SSEA-3 and CD105, rat anti-SSEA-3 (MA1020, Invitrogen™, Thermo Fisher Scientific, USA) and rabbit anti-CD105 (bs-4609-R, Bioss Antibodies, USA), were diluted 1:100 in PBS + 1% BSA and coverslips were incubated in a humid chamber for 16 hours at 4°C.

Next, coverslips were washed three times with PBS and incubated with the secondary antibodies diluted 1:1000 in PBS with 1% BSA in a dark humid chamber for 45 minutes at room temperature. Secondary antibodies were Alexa Fluor™ 568 anti-rat (A-11077, Invitrogen™, Thermo Fisher Scientific, USA) and Alexa Fluor™ 488 anti-rabbit (A-11008, Invitrogen™, Thermo Fisher Scientific, USA). After three PBS washes, coverslips were mounted with Fluoromount-G™ with DAPI (00-4959-52, Invitrogen™, Thermo Fisher Scientific, USA). Microphotographs were obtained using an Olympus BX-61 fluorescence microscope.

### 2.7 Immunocytofluorescence analysis of CD105 and SSEA-3 in canine and feline ADSCs

ADSCs were seeded onto coverslips (Mattek, USA) until 50–60% confluence and then washed with PBS 1X (HyClone™, Cytiva, USA) and fixed with 4% paraformaldehyde (Thermo Fisher Scientific, USA) for 30 minutes at room temperature. After three PBS 1X washes, coverslips were blocked with PBS 1X + 3% BSA (Sigma-Aldrich®, Germany) for 10 min.

Primary antibodies against SSEA-3 and CD105, rat anti-SSEA-3 (MA1020, Invitrogen™, Thermo Fisher Scientific, USA) and rabbit anti-CD105 (bs-4609-R, Bioss Antibodies, USA), were diluted 1:100 in PBS 1X + 3% BSA and coverslips were incubated in a dark humid chamber overnight at 4°C.

Next, coverslips were washed three times with PBS 1X and incubated with the secondary antibodies diluted 1:1000 in PBS 1X + 3% BSA in a dark chamber for 1 hour at room temperature. Secondary antibodies were Alexa Fluor™ 568 anti-rat (A-11077, Invitrogen™, Thermo Fisher Scientific, USA) and Alexa Fluor™ 488 anti-rabbit (A-11008, Invitrogen™, Thermo Fisher Scientific, USA)

After three PBS 1X washes, coverslips were mounted using Fluoromount-G™ with DAPI (00-4959-52, Invitrogen™, Thermo Fisher Scientific, USA). Microphotographs were obtained using a Nikon Eclipse Ts2R-FL fluorescence microscope equipped with a Nikon DS-Fi3 camera and Nis-Elements imaging software (Version 5.21.03).

### 2.8 **Immunomagnetic rabbit SSEA**-**3⁺/CD105⁺ cells purification using Magnetic-activated cell sorting (MACS)**

The ADSC culture from a single rabbit (n = 1) was expanded in high-glucose DMEM (HyClone™, Cytiva, USA) supplemented with 10% FBS (Gibco™, Thermo Fisher Scientific, USA), 2 mM glutamine (Sigma-Aldrich®, Germany), and 1% penicillin/streptomycin/amphotericin B (HyClone™, Cytiva, USA).

Immunomagnetic separation of SSEA-3⁺/CD105⁺ cells was performed using the MACS® MicroBeads cell isolation system (Miltenyi Biotec, Germany) according to the manufacturer’s instructions. ADSCs were subjected to sequential purification: the first step isolated the SSEA-3⁺ cell population, followed by a second step to isolate CD105⁺ cells from the SSEA-3⁺ population, thereby obtaining the double-positive SSEA-3⁺/CD105⁺ cells.

For SSEA-3⁺ isolation, ADSCs were suspended in MACS buffer, containing 0.5% BSA (Sigma-Aldrich®, Germany) and 2 mM EDTA (Sigma-Aldrich®, Germany) in PBS (HyClone™, Cytiva, USA), and were incubated with rat anti-SSEA-3 antibody (MA1020, Invitrogen™, Thermo Fisher Scientific, USA) for 15 minutes on ice. After three washes with MACS buffer and centrifugation at 300 g for 10 minutes, cells were incubated with the secondary antibody, anti-rat Kappa MicroBeads (130-047-401, Miltenyi Biotec, Germany), for 15 min on ice and washed three more times. Cells were resuspended in 500 µL MACS buffer and loaded onto MS columns for magnetic-activated cell sorting (MACS).

SSEA-3⁺ cells were suspended in MACS buffer and incubated with rabbit anti-CD105 antibody (bs-4609-R, Bioss Antibodies, USA) for 30 min on ice, washed three times with MACS buffer, and centrifuged at 300g for 10 minutes. Cells were then incubated with the secondary antibody, anti-rabbit IgG MicroBeads (130-048-602, Miltenyi Biotec, Germany), for 15 min on ice and washed three more times. Cells were resuspended in 500 µL MACS buffer and loaded onto MS columns for MACS.

Purified SSEA-3⁺/CD105⁺ cells were resuspended in DMEM supplemented with 10% FBS and 1% penicillin/streptomycin/amphotericin B and cultured at 37°C in a humidified atmosphere with 5% CO₂. SSEA-3⁺/CD105⁺ cells were plated on 75 cm² cell culture flasks (Nunc™, Thermo Fisher Scientific, USA) and subjected to further analysis.

These MACS-enriched SSEA-3⁺/CD105⁺ cells were used exclusively for descriptive analyses of Nanog, Sox2, and Oct3/4 expression.

### 2.9 **Immunocytofluorescence analysis of Sox2, Nanog, and Oct3/4 in rabbit SSEA**-**3⁺/CD105⁺ cells**

For immunocytofluorescence analysis, purified SSEA-3⁺/CD105⁺ cells obtained from a single rabbit (n = 1) were seeded onto coverslips (Mattek, USA) reaching 50–60% confluence and then washed with PBS 1X (HyClone™, Cytiva, USA) and fixed with 4% paraformaldehyde (Thermo Fisher Scientific, USA) for 30 min at room temperature. After washing three times with PBS 1X, coverslips were permeabilized with 0.1% Triton X-100 (Roche Diagnostics, Germany) in PBS 1X for 15 min with agitation and subsequently blocked with PBS 1X + 3% BSA (Sigma-Aldrich®, Germany) for 10 minutes.

Coverslips were then incubated overnight with primary antibodies diluted 1:100 in PBS 1X + 3% BSA in a dark, humid chamber at 4°C. The primary antibodies used were mouse anti-Sox2 (MA1-014, Invitrogen™, Thermo Fisher Scientific, USA), mouse anti-Nanog (14-5768-82, Invitrogen™, Thermo Fisher Scientific, USA), and rat anti-Oct3/4 (14-5841-82, Invitrogen™, Thermo Fisher Scientific, USA).

After incubation, coverslips were washed three times with PBS 1X and incubated with the secondary antibodies Alexa Fluor™ 568 anti-rat (A-11077, Invitrogen™, Thermo Fisher Scientific, USA) and Alexa Fluor™ 488 anti-mouse (A-11029, Invitrogen™, Thermo Fisher Scientific, USA) diluted 1:1000 in PBS 1X + 3% BSA in a dark chamber for 1 hour at room temperature.

Finally, coverslips were washed three times with PBS 1X and mounted using Fluoromount-G™ with DAPI (00-4959-52, Invitrogen™, Thermo Fisher Scientific, USA). Microphotographs were obtained using a Nikon Eclipse Ts2R-FL fluorescence microscope equipped with a Nikon DS-Fi3 camera and Nis-Elements imaging software (Version 5.21.03).

### 2.10 Immunofluorescence quantification and statistical analysis

Immunofluorescence images were analyzed using ImageJ software (National Institutes of Health, USA) by overlaying the blue, red, and green channels and manually quantifying marker-positive and marker-negative cells. Cells were considered positive when a DAPI-positive cell exhibited a specific red or green, fluorescent signal corresponding to the expected cellular localization of each marker (cell surface-associated for SSEA-3 and CD105; and nuclear for Nanog, Oct3/4, and Sox2) and clearly distinguishable from background fluorescence. No automated thresholding or segmentation procedures were applied. Image quantification was performed under blinded conditions, with the evaluator unaware of the species and sample identity. For each sample, five non-overlapping microscopic fields were analyzed for SSEA-3 and CD105 staining, whereas six non-overlapping microscopic fields were analyzed for Nanog, Oct3/4, and Sox2 expression. A total of 2035, 1526 and 1214 cells were evaluated for SSEA-3/CD105 quantification in rabbit, dog and cat, respectively; and 708, 696 and 714 cells were evaluated for Nanog, Oct3/4, and Sox2 expression, respectively. The percentage of positive cells was calculated for each field, and the mean value across all analyzed fields from each animal was used as a single biological replicate for subsequent statistical analyses. Thus, the animal was considered the experimental unit. SSEA-3/CD105 quantification was performed in samples from seven rabbits, three dogs, and three cats. Nanog, Oct3/4, and Sox2 expression was assessed in a single rabbit sample following MACS-based cell enrichment and was therefore evaluated descriptively only. Specificity of immunofluorescence staining was assessed using negative controls incubated with the secondary antibody alone, without the primary antibody (Supplementary Fig. 1). SSEA-3, CD105, Nanog, Oct3/4, and Sox2 antibodies were selected based on manufacturer-reported reactivity and their previous use in stem-cell characterization studies across multiple mammalian species. However, formal species-specific validation in rabbit, canine, and feline cells was beyond the scope of the present study.

Statistical analyses were performed exclusively to compare the percentage of SSEA-3⁺/CD105⁺ cells among the three species. Data distribution was assessed using the Shapiro–Wilk test, and homogeneity of variances was evaluated using Levene’s test. Although the data followed a normal distribution, the assumption of homogeneity of variances was not met. Due to the limited sample size and the unequal variances among groups, non-parametric analyses were performed. Differences among species were assessed using the Kruskal–Wallis test for independent samples with Bonferroni-adjusted pairwise comparisons when appropriate. Statistical analyses and graphical representations were performed using IBM SPSS Statistics version 29 (IBM Corp., USA). A p-value < 0.05 was considered statistically significant.

Unless otherwise indicated, data are presented as mean ± standard deviation (SD) calculated from independent biological replicates (animals), and range (minimum–maximum). For analyses performed on the single MACS-purified rabbit sample, values are presented as mean ± SD of the analyzed microscopic fields and are provided for descriptive purposes only.

## 3 Results

Immunocytofluorescence analysis evaluated CD105 and SSEA-3 expression in ADSCs from rabbits, dogs, and cats, as well as Nanog, Oct3/4, and Sox2 expression in purified rabbit SSEA-3⁺/CD105⁺ cells.

### 3.1 Rabbit

In rabbit ADSCs (n = 7), SSEA-3⁻/CD105⁺ cells represented 23.14 ± 7.15% (16.20–34.51), while no SSEA-3⁺/CD105⁻ cells were detected. The SSEA-3⁺/CD105⁺ population accounted for 54.84 ± 7.34% (43.90–61.97), and SSEA-3⁻/CD105⁻ cells represented 22.02 ± 5.00% (16.52–28.97). Representative images and the corresponding quantitative plot are shown in Fig. 1.

**Fig. 1:**
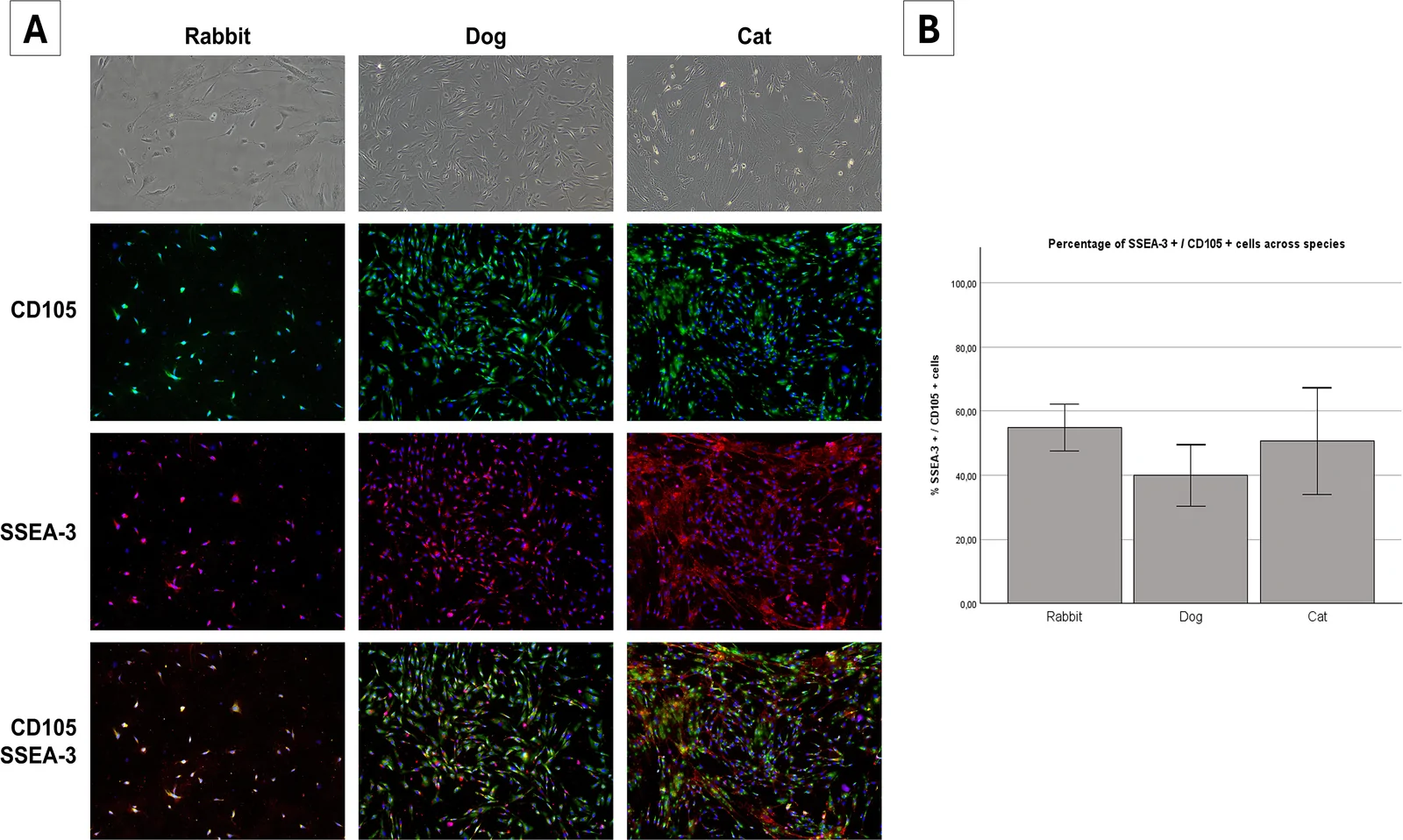
A) Immunocytofluorescence characterization of ADSCs from rabbit, dog, and cat adipose tissue. The top row shows bright-field images of the cell cultures, while the following rows depict the immunocytofluorescence staining. Nuclei appear stained with DAPI (blue). CD105+ cells are shown in green, and SSEA-3+ cells are shown in red. The last row displays the double-positive SSEA-3⁺/CD105⁺ cells. All images were acquired using a 10x objective. B) Quantification of the SSEA-3⁺/CD105⁺ cell population identified in rabbit (n = 7), dog (n = 3), and cat (n = 3) ADSCs. Bar graphs represent the percentage of double-positive CD105⁺/SSEA-3⁺ cells obtained by immunocytofluorescence analysis. Error bars represent the SD calculated from independent biological replicates (animals).

After immunomagnetic purification, the proportion of SSEA-3⁺/CD105⁺ cells in the single rabbit sample subjected to MACS was 88.36 ± 0.98% (SD among analyzed microscopic fields), compared with 54.84 ± 7.34% in the non-purified samples (SD among individual animals; n = 7 rabbits). These findings are consistent with an apparent enrichment of this cell population following MACS in the analyzed rabbit sample. In the MACS-purified rabbit SSEA-3⁺/CD105⁺ cell population (n = 1 rabbit), immunocytofluorescence showed high expression of the pluripotency-associated transcription factors Nanog+ (87.65 ± 5.16%), Oct3/4+ (88.96 ± 1.55%), and Sox2+ (89.15 ± 2.84%), with values expressed as mean ± SD of analyzed microscopic fields. Representative images and quantitative plots are shown in Fig. 2.

**Fig. 2:**
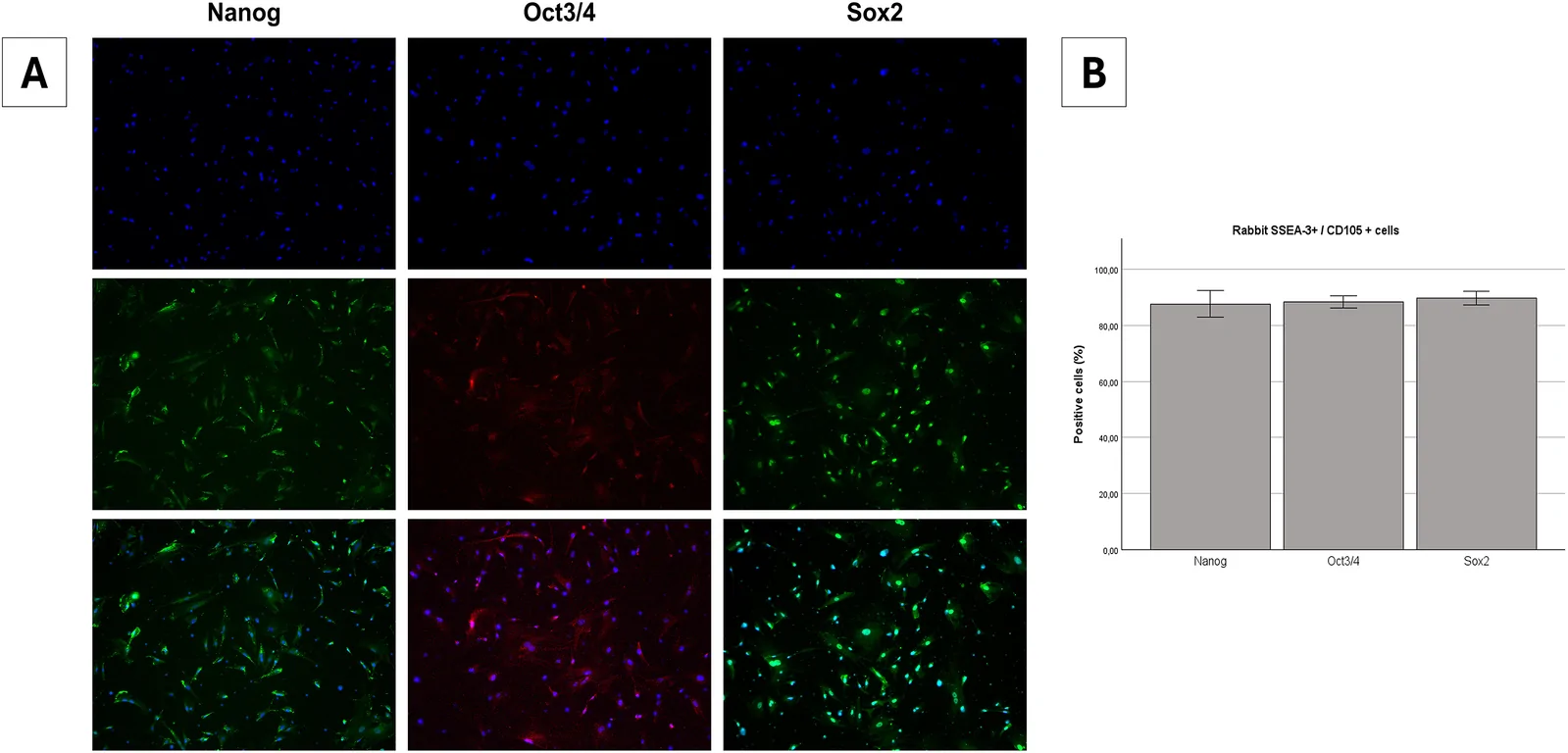
A) Immunocytofluorescence characterization of purified rabbit SSEA-3⁺/CD105⁺ cells derived from adipose tissue. The first row shows nuclei stained with DAPI (blue). The next row shows immunofluorescence staining of Nanog+ (green), Oct3/4+ (red), and Sox2+ (green) cells. The bottom row displays merged images of DAPI with each transcription factor. All images were acquired using a 10x objective. B) Quantification of Nanog, Oct3/4 and Sox2 expression in purified rabbit SSEA-3⁺/CD105⁺ cells derived from adipose tissue (n = 1). Bar graphs represent the percentage of positive cells obtained by immunocytofluorescence analysis. Error bars represent the standard deviation among analyzed microscopic fields and are provided for descriptive purposes only.

### 3.2 Dog

In dog ADSCs (n = 3 animals), SSEA-3⁻/CD105⁺ cells represented 53.15 ± 11.27% (40.73–62.71), while SSEA-3⁺/CD105⁻ cells accounted for 4.61 ± 2.16% (2.15–6.19). The SSEA-3⁺/CD105⁺ subset comprised 39.93 ± 9.58% (29.93–49.02), and SSEA-3⁻/CD105⁻ cells 2.31 ± 1.58% (0.98–4.06). Representative images and the corresponding quantitative plot are shown in Fig. 1.

### 3.3 Cat

In cat ADSCs (n = 3 animals), SSEA-3⁻/CD105⁺ cells represented 30.40 ± 17.51% (10.33–42.51), SSEA-3⁺/CD105⁻ cells 13.77 ± 1.89% (12.41–15.93), SSEA-3⁺/CD105⁺ 50.61 ± 16.67% (39.31–69.75), and SSEA-3⁻/CD105⁻ cells 5.21 ± 1.06% (3.99–5.89). Representative images and the corresponding quantitative plot are shown in Fig. 1.

Comparative statistical analysis revealed no significant differences in the percentage of SSEA-3⁺/CD105⁺ cells among the three species. Table 1 summarizes the relative abundance of CD105⁺/SSEA-3⁺ cells in rabbit, dog, and cat samples, alongside other subpopulations for comparison.

**Table 1:** Summary of CD105 and SSEA-3 expression in ADSCs from rabbit, dog and cat.

**Species**	**ADSC subpopulations**			
	**SSEA-3⁻/CD105⁺ (%)**	**SSEA-3⁺/CD105⁻ (%)**	**SSEA-3⁺/CD105⁺ (%)**	**SSEA-3⁻/CD105⁻ (%)**
**Rabbit** (n=7)	23.14 ± 7.15 (16.20–34.51)	0	54.84 ± 7.34 (43.90–61.97)	22.02 ± 5.00 (16.52–28.97)
**Dog** (n=3)	53.15 ± 11.27 (40.73–62.71)	4.61 ± 2.16 (2.15–6.19)	39.93 ± 9.58 (29.93–49.02)	2.31 ± 1.58 (0.98–4.06)
**Cat** (n=3)	30.40 ± 17.51 (10.33–42.51)	13.77 ± 1.89 (12.41–15.93)	50.61 ± 16.67 (39.31–69.75)	5.21 ± 1.06 (3.99–5.89)

Values represent the percentage of each subpopulation defined by CD105 and SSEA-3 expression: SSEA-3⁻/CD105⁺, SSEA-3⁺/CD105⁻, SSEA-3⁺/CD105⁺, and SSEA-3⁻/CD105⁻. Data are shown as mean ± standard deviation (SD), with the range (minimum–maximum) in parentheses.

## 4 Discussion

In this study, ADSCs from rabbit, dog, and cat adipose tissue were characterized by immunocytofluorescence for CD105 and SSEA-3, identifying an SSEA-3⁺/CD105⁺ cell population exhibiting immunophenotypic characteristics consistent with a Muse-like population in all three species, although functional characterization was beyond the scope of the present study. Rabbit SSEA-3⁺/CD105⁺ cells were additionally purified by MACS and analyzed for Sox2, Nanog, and Oct3/4 expression. The proportion of SSEA-3⁺/CD105⁺ cells varied among species, although no statistically significant differences were detected. In addition, the purified rabbit SSEA-3⁺/CD105⁺ cell population exhibited high expression of the pluripotency-associated markers Nanog, Oct3/4, and Sox2. These findings provide comparative immunophenotypic data for rabbit, canine, and feline ADSCs based on SSEA-3 and CD105 expression. However, the additional characterization of Nanog, Oct3/4, and Sox2 expression was performed exclusively in rabbit SSEA-3⁺/CD105⁺ cells. Therefore, the depth of characterization was not equivalent among species and comparisons regarding pluripotency-associated marker expression cannot be extended to dogs or cats.

### 4.1 CD105 and SSEA-3

CD105, also known as endoglin, is a membrane glycoprotein abundantly expressed on mesenchymal stem cells (MSCs)(Lin et al., 2013; Pittenger et al., 1999), while SSEA-3 is a cell surface glycosphingolipid typically used as a marker of pluripotent stem cells, such as ESCs(Thomson et al., 1998). Muse cells can be isolated from MSCs based on their double positivity for SSEA-3 and CD105. These cells exhibit self-renewal capacity, non-tumorigenicity, and trilineage differentiation potential (Kuroda et al., 2010).

Besides its use as a marker for Muse cell identification, SSEA-3 may also play a biological role in stem cell regulation. Previous studies have suggested that SSEA-3 is involved in FGF2-dependent self-renewal, clonogenicity, maintenance of stemness, and lineage commitment, indicating that this glycolipid may contribute to the biological properties of pluripotent-like stem cell populations rather than functioning solely as a passive surface marker (Aprile et al., 2023; Lee et al., 2025).

Importantly, SSEA-3 is a glycosphingolipid rather than a protein epitope, a biochemical feature that ensures high cross-species conservation. Wakao et al. (2026) highlighted that this molecular nature allows anti-SSEA-3 antibodies to reliably detect Muse cells across mammals, supporting the applicability of an SSEA-3-based detection strategy in rabbits, dogs, and cats.

Over the past 15 years, several studies have quantified Muse cells across different species and tissue sources, generally reporting low percentages. In humans, Muse cells account for approximately 1.1 ± 0.05% of bone marrow-derived mesenchymal stem cells (BMSCs) and 1.8 ± 0.22% of fibroblasts(Kuroda et al., 2010). When isolated from ADSCs, the proportion increases, ranging from 1.91 ± 0.42% to 8.8 ± 1.3%(Kinoshita et al., 2015; Ogura et al., 2014), with intermediate values, such as 2.2 ± 1.1% and 3.2 ± 0.7%, also reported (Fouad et al., 2018; Yamauchi et al., 2017). In rabbits, Yamada et al. (2018) reported about 0.5% of SSEA-3⁺ cells among BMSCs, whereas in rodents, values of 3.0% in mice(Aprile et al., 2021) and 2.3 ± 0.3% in rats(Sun et al., 2020) have been described. In swine, approximately 2% of Muse cells have been identified among BMSCs(Iseki et al., 2021), while goat SSEA-3⁺ cells accounted for nearly 4% of fibroblasts(Yang et al., 2013), and horse Muse cells represented 2.1 ± 0.7% of BMSCs(Lee et al., 2025). Regarding ADSCs, the proportion of mouse Muse cells ranged from 2.6%(Aprile et al., 2021) to 6.3 ± 1.9%(Nitobe et al., 2019), while sheep exhibited a markedly elevated percentage of Muse cells, reaching 15.6 ± 2.9%(Castillo et al., 2023). Overall, variability between species and tissues is evident, with adipose samples tending to show higher values compared to other sources. The first report in dogs(Mitani et al., 2021) did not identify “classical” Muse cells. Instead, it described “long-term trypsin treatment (LTT)–tolerant cells,” which represented 9.3 ± 0.5% of ADSCs. Although these cells shared functional properties with human Muse cells, their phenotype differed substantially, limiting direct comparisons. Recently, the presence of Muse cells has been estimated in umbilical cord derived MSCs (UC-MSCs) and in ADSCs from dogs and cats. While the first study reported 1.12% of SSEA-3⁺ cells in feline UC-MSCs and 0.14% in canine UC-MSCs(Chen et al., 2026); the second one shows slightly higher percentages of SSEA-3^+^ cells in ADSCs, accounting for 2.9 ± 0.15% in cats and 0.93 ± 0.16% in dogs(Wakao et al., 2026).

In contrast to previous reports, the present study found substantially higher percentages of SSEA-3⁺/CD105⁺ cells in rabbit, dog, and cat ADSCs, with values of 54.84 ± 7.34%, 39.93 ± 9.58%, and 50.61 ± 16.67%, respectively. These values exceed those reported in most previous studies, highlighting the need for cautious interpretation and independent validation. Although the factors responsible for these differences were not directly investigated, both biological and methodological variables may have contributed to the observed proportions.

Several factors may have contributed to the high proportion of SSEA-3⁺/CD105⁺ cells observed in this study, with tissue source being particularly relevant. In mice, for example, Muse cell percentages vary depending on the tissue: 3.0% in BMSCs, 2.6% in ADSCs, and 2.1% in fibroblasts(Aprile et al., 2021). Within this context, depot-dependent differences in yield, viability, and differentiation capacity of ADSCs have been reported in human adipose tissue(Russo et al., 2014), suggesting that not all fat depots are biologically equivalent.

In the present study, the adipose tissue depot differed among species due to differences in tissue availability during sample collection. Specifically, samples were collected from the inguinal depot in rabbits, the falciform ligament in dogs, and mesenteric adipose tissue in cats. Therefore, the variability observed among species may reflect a combination of species-related biological variability and depot-specific properties, although these factors were not directly evaluated in the present study.

Furthermore, in the previous report of Muse cells in canine and feline ADSCs(Wakao et al., 2026), adipose tissue was collected from the uterine broad ligament and subcutaneous tissue. Differences between adipose depots and sampling locations appear to be a relevant source of variability in Muse-like cell yield. Each depot presents a distinct microenvironment—defined by its vascularization, inflammatory status, degree of hypoxia, and stromal composition—and visceral or mesenteric fat typically exhibits higher oxidative and mechanical stress, favouring stress-tolerant subpopulations such as Muse cells(Ong et al., 2021). These anatomical and physiological differences may influence the detection and abundance of SSEA-3⁺/CD105⁺ cells and could contribute to variability among studies. Moreover, Wakao et al. (2026) noted that canine and feline populations display substantial genetic diversity and frequent hybridization, which may further contribute to interindividual and interstudy variability in SSEA-3^+^/CD105^+^ cell yield.

Other factors such as donor characteristics or culture conditions likely contribute to the observed variability. Although the impact of donor-related variables such as age, sex, or body mass index (BMI) on Muse-like cell yield and viability may be remarkable, only one study addresses this issue. Yamauchi et al. (2017) found an inverse correlation between donor age and the number of SSEA-3⁺ cells per gram of fat, indicating that aging may negatively affect Muse cell abundance, particularly in adipose-derived sources. However, no additional studies have directly evaluated the influence of age, sex, or BMI on Muse cells, but evidence from MSC research suggests similar trends.

For instance, Varghese et al. (2017) reported that increased donor age is associated with reduced stem cell yield, proliferation rate, and differentiation potential. Likewise, elevated BMI has been linked to impaired ADSC functionality, including reduced proliferation and differentiation capacity. In contrast, sex does not appear to significantly affect adipocyte precursor cells yield or proliferation(van Harmelen et al., 2004). It is reasonable to hypothesize that SSEA-3^+^/CD105^+^ cells, as a subpopulation of MSCs, may also be affected by donor-dependent factors. Although these findings have not yet been confirmed in Muse cells, existing evidence suggests these factors could influence both isolation efficiency and regenerative potential, emphasizing the need for further research.

Culture conditions are another critical variable. The composition of the basal medium, supplements—such as serum type, and environmental factors—such as oxygen tension—, can significantly alter the cellular heterogeneity of ADSCs(Baer & Geiger, 2012). Given that Muse cells are more stress-tolerant than MSCs(Kuroda et al., 2010), it is plausible that more demanding culture conditions may favor their survival and enrichment. Supporting this, Li et al. (2025) demonstrated that culturing BMSCs under 1% O₂ hypoxia nearly doubled the proportion of Muse cells (from 3.9 ± 0.68% to 7.9 ± 1.34%) and enhanced the expression of pluripotency genes. Although hypoxic conditions were not applied in the present study, this example illustrates how culture parameters can influence Muse-like cells quantification and may partly explain discrepancies between studies employing different protocols. Standardizing ADSCs culture conditions, particularly oxygen tension and medium composition, may therefore be essential to improve reproducibility in Muse cell research.

Passage number and cell density also influence subpopulation dynamics. Early-passage ADSCs exhibit considerable heterogeneity, which tends to decrease with successive passages, resulting in a more homogeneous population(Peng et al., 2020). Although SSEA-3 expression was not specifically assessed in that study, other markers showed significant shifts across passages, indicating changes in subpopulation composition. Moreover, Kuroda et al. (2010) reported that Muse cells maintain viability through successive subcultures, with a nearly twofold increase in Muse cell clusters from passage 2 to 5, likely due to their stress resistance. Nevertheless, a decline in the percentage of SSEA-3⁺ cells at higher passages (≥10) has also been documented (Kuroda et al., 2013).

Other methodological variables related to marker detection may also contribute to the observed proportions of SSEA-3⁺/CD105⁺ cells. Variations in antibody specificity, staining protocols, and fluorochrome sensitivity may influence quantification, as even small differences in reagents or culture media affect marker detection(Tatsumi et al., 2018). The absence of species-specific antibody validation and additional controls should be considered when interpreting these findings, as potential differences in antibody specificity, cross-reactivity, or performance among species cannot be completely excluded. Furthermore, when immunofluorescence-based analysis is performed, differences in quantification criteria, fluorescence thresholds, and image analysis procedures may also contribute to variability among studies. Therefore, standardization of staining procedures, quantification criteria, and isolation approaches is essential for meaningful cross-study comparisons. Finally, differences in quantification, isolation, or enrichment techniques—such as MACS, fluorescence-activated cell sorting (FACS), or LTT—may further contribute to variability(Li et al., 2022; Uchida et al., 2017). Thus, the reported proportions should be interpreted considering these methodological considerations until further validation studies are performed.

In summary, although this study identified high percentages of SSEA-3⁺/CD105⁺ cells exhibiting immunophenotypic characteristics consistent with a Muse-like population in rabbit, dog, and cat adipose tissue, the findings should be interpreted with caution. Both biological factors, including species, adipose tissue source, donor characteristics, and culture conditions, and methodological factors, including antibody performance, immunofluorescence-based quantification, and image analysis procedures, may have contributed to the observed frequencies. However, since these variables were not directly investigated, their respective contributions cannot be determined, and the proposed explanations should be considered exploratory rather than definitive.

### 4.2 Nanog, Oct3/4 and Sox2

The transcription factors Nanog, Oct3/4, and Sox2 are well-established markers of pluripotency, essential for maintaining the undifferentiated state and self-renewal capacity in ESCs(Kashyap et al., 2009; Lee et al., 2010; Mitsui et al., 2003). Their expression has also been consistently demonstrated in human Muse cells using RT-PCR and immunofluorescence(Fouad et al., 2018; Kinoshita et al., 2015; Kuroda et al., 2010; Ogura et al., 2014; Wakao et al., 2011). Although positivity rates are not always reported, Muse cells generally show lower expression levels of these markers compared to ESCs and iPSCs, which aligns with their biological profile: pluripotent but non-tumorigenic(Kuroda et al., 2010; Wakao et al., 2011).

In the present study, the purified rabbit SSEA-3⁺/CD105⁺ cell population (n = 1 rabbit) exhibited high expression of all three transcription factors, with Nanog⁺ cells accounting for 87.65 ± 5.16%, Oct3/4⁺ for 88.96 ± 1.55%, and Sox2⁺ for 89.15 ± 2.84%. These values are presented descriptively and represent variability among analyzed microscopic fields.

Published reports show that the frequency of Muse cells positive for these transcription factors varies depending not only on the tissue source, but also across species. Aprile et al. (2021) reported that in mouse Muse cells derived from BMSCs and fibroblasts, Oct3/4 and Sox2 were expressed in over 75% of Muse cells, while Nanog was detected in approximately 70% and 50%, respectively. In Muse cells isolated from mouse ADSCs, Oct3/4 positivity reached nearly 80%, whereas Sox2 and Nanog were expressed in around 60% and 30% of cells. Similarly, Nitobe et al. (2019) confirmed the presence of all three markers in Muse cell clusters from mouse adipose tissue. In rat, Muse cells derived from BMSCs also expressed Nanog, Sox2, and Oct3/4, although specific percentages were not reported(Sun et al., 2020).

In equine SSEA-3+ cells derived from BMSCs, immunofluorescence analysis revealed lower expression for Nanog (19.8 ± 3.8%) and Oct3/4 (9.7 ± 2.6%), while Sox2 was highly expressed (97.3 ± 4.1%)(Lee et al., 2025). In other species, such as rabbit, RT-PCR has confirmed the expression of Nanog and Oct3/4(Yamada et al., 2018), and the presence of all three markers has been demonstrated in goat, swine and ovine(Castillo et al., 2023; Iseki et al., 2021; Yang et al., 2013). Consistent with this, Wakao et al. (2026) showed that canine and feline SSEA-3⁺ cells express significantly higher levels of Oct3/4 and Nanog compared with SSEA-3⁻ ADSCs and undergo spontaneous trilineage differentiation without cytokine induction, supporting the expression of pluripotency-associated markers in SSEA-3⁺ cell populations across different species.

Compared to these findings, the expression levels observed in the rabbit SSEA-3⁺/CD105⁺ cell population analyzed in the present study are among the highest reported, particularly for Nanog and Oct3/4. Potential explanations include species-specific differences, the use of MACS-enriched SSEA-3⁺/CD105⁺ cells, adipose tissue source, or other methodological factors; however, these possibilities were not directly investigated in the present study. However, these observations should be interpreted considering certain methodological limitations, since species-specific validation of the antibodies employed for immunofluorescence was not performed and positive and isotype controls were not included. These findings indicate that the rabbit SSEA3⁺/CD105⁺ cell population analyzed in this study exhibited high expression of pluripotency-associated markers. However, the expression of Nanog, Oct3/4, and Sox2 alone does not constitute functional evidence of pluripotency, and additional functional studies would be required to more conclusively characterize the biological properties of this cell population.

Finally, this expression is modulated by various environmental and methodological factors, such as hypoxia. For example, Li et al. (2025) showed that culturing Muse cells under hypoxic conditions (1% O₂) significantly upregulated Nanog, Sox2, and Oct3/4 expression compared to normoxia, indicating that stress-related environments can enhance the expression of pluripotency markers. These findings suggest that culture-induced stress may influence SSEA-3⁺ cell survival and transcriptional activation, although this possibility was not evaluated in the present study. Methodological consistency is therefore essential to ensure accurate cross-study comparisons and to avoid misinterpreting differences that may arise from culture dependent modulation rather than true biological variation.

### 4.3 Study limitations and future perspectives

Despite the strengths of the study, some limitations should be acknowledged. First, the number of biological samples was limited, especially for MACS enrichment and Nanog, Sox2 and Oct3/4 analysis. Whereas for the identification of the SSEA-3⁺/CD105⁺ population samples were obtained from 7 rabbits, 3 dogs and 3 cats, MACS only was performed in one of the rabbit samples. Therefore, further studies including a larger number of biological replicates will be required to confirm and extend these findings.

A limitation of this study is that species-specific validation of the antibodies used for immunofluorescence was not performed. Antibodies were selected based on manufacturer-reported reactivity and their previous use in stem-cell characterization studies across multiple mammalian species. In addition, SSEA-3 is a glycolipid epitope that has been reported to be highly conserved among mammalian species and has been successfully used for Muse cell identification in multiple animal models. Although negative controls consisting of secondary antibody alone showed no detectable staining, positive controls and isotype controls were not included. Therefore, contributions from nonspecific binding, antibody cross-reactivity, or species-related differences in antibody recognition cannot be completely excluded. Consequently, the immunofluorescence-based quantification of SSEA-3, CD105, Nanog, Oct3/4, and Sox2 expression should be interpreted with care, as the reported percentages may have been influenced by these methodological limitations. Further studies incorporating species-specific antibody validation and additional control experiments will be necessary to confirm the specificity of marker detection and the frequency of positive cells in these species.

Furthermore, the expression of Nanog, Sox2, and Oct3/4 was evaluated exclusively in the MACS-enriched rabbit SSEA-3⁺/CD105⁺ population. Therefore, no conclusions regarding the expression of these pluripotency-associated markers in canine or feline SSEA-3⁺/CD105⁺ cells can be drawn from the present study.

In addition, although SSEA-3 has been widely used for Muse cell isolation (Kuroda et al., 2010), the expression of SSEA-3 and CD105 alone does not demonstrate the complete functional identity of Muse cells. Therefore, the SSEA-3⁺/CD105⁺ cells identified in the present study should be considered a Muse-like population enriched based on immunophenotypic characteristics. Functional assays, including stress tolerance, self-renewal capacity, spontaneous trilineage differentiation, and migration toward injury signals, would be required to confirm their definitive functional identity as Muse cells (Kuroda et al., 2010). Likewise, although Nanog, Oct3/4, and Sox2 are widely recognized as pluripotency-associated markers, their expression alone does not demonstrate functional pluripotent capacity. Functional differentiation assays would be necessary to establish pluripotent characteristics more conclusively.

It should also be noted that SSEA-3 expression alone has been widely used for Muse cell isolation in several previous studies (Kuroda et al., 2010; Wakao et al., 2011). Therefore, the use of CD105 as an additional inclusion criterion in the present study may represent a more restrictive approach and may not encompass all SSEA-3⁺ cells potentially exhibiting Muse-like characteristics. While most Muse cells are expected to express mesenchymal markers such as CD105, the presence of SSEA-3⁺/CD105⁻ cells observed in some samples suggests that a subset of potentially relevant cells may have been excluded from the present analysis. Consequently, the reported SSEA-3⁺/CD105⁺ population should be considered a specifically selected subpopulation rather than the entirety of the SSEA-3⁺ compartment.

Another limitation is related to the use of adipose tissue from different anatomical locations among species. Differences in adipose depots may influence the cellular composition of the cultures and the expression of surface markers (Ong et al., 2021). Consequently, comparisons among species should be interpreted considering that samples were not obtained from identical anatomical sites.

The use of immunofluorescence for marker detection should also be considered when interpreting the frequency of SSEA-3⁺/CD105⁺ cells. This technique enabled the evaluation of marker co-expression at the single-cell level and the visualization of positive cells within the culture. However, immunofluorescence and flow cytometry rely on different analytical approaches and may differ in sensitivity, the number of cells analyzed, and quantitative assessment, which may influence the estimated proportion of marker-positive cells. Although immunofluorescence enabled evaluation of marker co-expression at the single-cell level, flow cytometry would allow analysis of substantially larger cell populations and a more standardized quantitative assessment. Therefore, future studies combining immunofluorescence with flow cytometry will be important to further validate the frequency of the SSEA-3+/CD105+ population reported here.

Overall, while further functional studies will be necessary to characterize the biological properties of this population, the present study provides relevant information on the identification and comparative characterization of an SSEA-3⁺/CD105⁺ cell population exhibiting immunophenotypic characteristics consistent with a Muse-like population in ADSCs from rabbit, dog and cat. Future studies will be required to confirm the biological identity and functional properties of these cells.

## 5 Conclusions

This study provides a comparative characterization of an SSEA-3⁺/CD105⁺ cell population in ADSCs from rabbits, dogs, and cats. The identification of this SSEA-3⁺/CD105⁺ immunophenotypic profile is consistent with the presence of a Muse-like population within the adipose tissue of these species. In rabbits, immunomagnetic isolation of SSEA-3⁺/CD105⁺ cells revealed expression of Nanog, Oct3/4, and Sox2 in the isolated population. However, expression of these markers alone does not demonstrate pluripotency, and further functional characterization will be required to establish whether these cells exhibit pluripotent characteristics. Accordingly, the comparative component of the present study is limited to the immunophenotypic characterization of the SSEA-3⁺/CD105⁺ population across the three species, whereas the assessment of pluripotency-associated markers was restricted to rabbit cells. Although differences among individuals, species, adipose tissue sources, culture conditions, antibody specificity, and methodological approaches for cell identification and quantification may influence the observed results and limit direct comparisons with previous studies, these findings expand current knowledge of SSEA-3⁺/CD105⁺ cell populations exhibiting immunophenotypic characteristics consistent with a Muse-like phenotype in veterinary species.

Overall, this study provides a basis for future investigations aimed at confirming the biological properties of this cell population, evaluating potential interspecies differences, and exploring its possible relevance in regenerative medicine.

## Ethics approval and consent to participate

All procedures were approved by the Animal Ethics and Experimentation Committee of CEU Cardenal Herrera University and by the competent authority (authorization 2023-VSC-PEA-0242). Owners of dogs and cats provided written informed consent.

## Consent for publication

Not applicable.

## Availability of data and materials

All data analysed during the current study are available from the corresponding author on reasonable request.

## Funding

This work was supported by the Generalitat Valenciana through the Emerging Research Groups Grant program (CIGE/2022/118), the Garcia Cugat Foundation CEU-UCH Chair and CEU Cardenal Herrera University (GIR25/60 and INDI25/77). M.G.V.-M. acknowledges support from a FPU predoctoral fellowship from the Spanish Ministry of Science, Innovation and Universities.

## Statement for studies in animals

All procedures were approved by the Animal Ethics and Experimentation Committee of CEU Cardenal Herrera University and by the competent authority (authorization 2023-VSC-PEA-0242). Owners of dogs and cats provided written informed consent.

## CRediT authorship contribution statement

**María Gemma Velasco-Martínez:** Writing – original draft, Methodology, Investigation, Formal analysis, Data curation, Conceptualization. **Belén Cuervo:** Writing – review & editing, Funding acquisition, Data curation, Conceptualization. **Emma Martins:** Methodology, Data curation. **Rocío Colomer-Selva:** Methodology, Data curation. **Maria Descalzo-Navarro:** Methodology, Formal analysis. **Beatriz Mena-Moros:** Methodology, Formal analysis. **Deborah Chicharro:** Writing – review & editing, Methodology. **José María Carrillo:** Writing – review & editing, Funding acquisition, Conceptualization. **Joaquín Jesús Sopena:** Writing – review & editing, Funding acquisition, Conceptualization. **Mónica Rubio:** Writing – review & editing, Methodology, Formal analysis. **Katiuska Satué:** Writing – review & editing, Writing – original draft, Formal analysis.

## Declaration of competing interest

The authors declare that they have no competing interests.
