# Supplementary material for: Identification and characterization of SSEA-3⁺/CD105⁺ cells in adipose tissue of rabbits, dogs, and cats: insights into a Muse-like cell population
Source: Vet Anim Sci. 2026 Oct 25;34:100917. doi: 10.1016/j.vas.2026.100917 (PMC13769589; doi:10.1016/j.vas.2026.100917)
Supplement: Supplementary file 1 [file mmc1.docx]

**Supplementary material**

Supplementary Figure 1. Negative controls for immunofluorescence staining. Representative images of negative controls in which primary antibodies were omitted and the corresponding secondary antibodies were included. No specific fluorescence signal was detected.
